# Supplementary material for: WSe2 Light-Emitting Device Coupled to an h-BN Waveguide
Source: ACS Photonics. 2023 Mar 23;10(5):1328–33. doi: 10.1021/acsphotonics.2c01963 (PMC10197165; doi:10.1021/acsphotonics.2c01963)
Supplement: Supplementary file 1 — ph2c01963_si_001.pdf [file ph2c01963_si_001.pdf]

Supporting Information: WSe_2 light-emitting device coupled to h-BN waveguide

Ronja Khelifa,[1,*] Shengyu Shan,[1,*] Antti J. Moilanen,[1] Takashi Taniguchi,[2] Kenji Watanabe,[3] and Lukas Novotny[1,†]
[1]*Photonics Laboratory, ETH Zürich, 8093 Zürich, Switzerland*
[2]*International Center for Materials Nanoarchitectonics, National Institute for Materials Science, 1-1 Namiki, Tsukuba 305-0044, Japan*
[3]*Research Center for Functional Materials, National Institute for Materials Science, 1-1 Namiki, Tsukuba 305-0044, Japan*
(Dated: March 14, 2023)

CONTENTS

I. Sample Fabrication S2
A. Stacking S2
B. Contacting S2
C. Patterning into a waveguide S2

II. Measurements S2
A. Optical Characterization S2
B. Electroluminescence S3

III. Efficiency calculation of the LEDs S3
A. Definition S3
B. Correction for the detection path S3
C. Results S3

IV. Photoluminescence measurements S4

V. Graphene edge contacts on thick stacks S4

VI. Band alignment of the tunneling junction S5

VII. Spectral characteristics of the waveguide-coupled emission S5

VIII. Details on the Coupling efficiency S6
A. Experiments S6
B. Simulations S7

References S8

* These two authors contributed equally
† Corresponding author, lnovotny@ethz.ch

I. SAMPLE FABRICATION

A. Stacking

Monolayer graphene (Gr), monolayer WSe_2, and multilayer hexagonal-Boron Nitride (h-BN) flakes were mechanically exfoliated on top of SiO_2/Si substrates. Monolayer Gr and WSe_2 were identified under an optical microscope and the h-BN flakes with desired thickness were selected by scans of an atomic force microscope (AFM). The van der Waals stack was assembled using a curved polydimethylsiloxane (PDMS) stamp covered with a polycarbonate (PC) film in a glovebox filled with Argon (Ar) gas [1, 2]. In order to see through the flakes during stacking for better alignment, a thin top h-BN, ∼5 nm was picked up as the first layer. In the following steps, this thin top h-BN was used to pick up a top Gr, a tunneling h-BN, a monolayer WSe_2, a second tunneling h-BN, a bottom Gr and a bottom thick h-BN (∼105 nm) sequentially. This stack then was transferred onto a glass coverslip. Finally, a thick h-BN flake (130 nm − 135 nm) was picked up with a new PC film. This thick h-BN was used as the top layer to pick up the previous stack. The final stack, consisting of the LED encapsulated in between thick h-BNs, was transferred to a glass substrate. After both transfers, the PC films were dissolved in chloroform for ∼1 hour at room temperature.

B. Contacting

The position of the Gr flakes was identified by performing a reflection map. We contacted the encapsulated Gr flakes following the Gr edge contact recipe described in Refs. [1, 3]. Electron-beam lithography (EBL) was used to define the pattern of the contacts. Then the stack was etched by reactive ion etching (RIE) with CHF_3 and O_2. After the etching, 10 nm chromium and 120 nm gold were evaporated as contact metals. The etching depth was chosen around 150 nm, so that the Gr edges were exposed while the gold remained continuous at the etched step.

C. Patterning into a waveguide

By measuring the electroluminescence (EL) of the device after contacting, the position of the overlap region was confirmed and the waveguide structure was designed accordingly. A layer of 450 nm-thick 950K PMMA was spin-coated as EBL resist followed by a layer of ESPACER 300Z to avoid charging. The designed pattern was exposed under 30 kV at a dose of 400 $\mu A/cm^2$ after proximity effect correction. The ESPACER 300Z was removed in de-ionized (DI) water within 2 min. The sample was developed for 90 s in a cold mixture of isopropanol (IPA) and DI water with a weight ratio of 3:1. The stack was etched in steps with RIE (SF_6 and Ar (20 sccm: 20 sccm), 95 W and 100 mTorr). The cross-linked PMMA at the surface was removed by RIE with O_2 (20 sccm, 60 W and 40 mTorr). Then the rest of the PMMA was removed in warm Acetone (40 min).

II. MEASUREMENTS

A. Optical Characterization

Photoluminescence (PL) and reflection characterization were performed after stacking in ambient conditions. The sample was positioned on an in-plane piezoelectric stage on a customized inverted Nikon TE300 microscope, pumped by a 532 nm solid state continuous wave (cw) laser for both measurements. The excitation power for PL is in the order of 1 μW. An oil-immersion objective (Nikon, 100x. NA 1.4) was used to focus the incident laser onto the sample as well as to collect the reflected and emitted light from the sample through the glass substrate. By scanning the piezoelectric stage, we could obtain a reflection map based on the photon counts at different positions. To record the PL signal, in addition, a long-pass filter was used to filter out the laser excitation. The PL spectra were acquired with a Princeton Instruments SpectraPro HRS-300 spectrometer with a BLAZE 400-HRX camera. The spectra were corrected to the wavelength-dependent transmission of the setup. Real space and Fourier space (back focal plane) images were recorded with an electron-multiplying charge-coupled device (EMCCD, Andor iXon Ultra). For the transmission measurement, the sample is illuminated by white light that is focused on the sample through an objective (Olympus, 10x, NA 0.3). The transmitted light is collected by a second objective (Nikon, 100x, NA 0.9) and wavelength-resolved by the spectrometer. As the broadband source, we use a halogen lamp that covers wavelengths from visible to near-infrared.

B. Electroluminescence

For the EL measurements (also in ambient conditions), the sample was positioned on the same piezo stage while no incident laser was needed. A bias voltage, provided by a Keithley 2602B source meter unit, was applied to the sample through probe needles and the emitted light could be detected with the same oil-immersion objective as used for PL. If necessary, an aperture was used to additionally block the light from a specific region. For spatially resolved spectral measurements, the slit opening at the entrance of the spectrometer was used to limit the range in the horizontal direction, and the integration over selected rows of pixels in post-processing limited the range in the vertical direction. Because the scattering at the edge of the LED region is very strong, additional background corrections were performed and measurements with spatially blocked LED emission were conducted. Also for EL the spectra were corrected by the wavelength-dependent transmission of the setup.

III. EFFICIENCY CALCULATION OF THE LEDS

A. Definition

The external quantum efficiency of the LED was defined and calculated as

$$\eta_{\mathrm{EQE}} = \frac{\#\ \mathrm{Photons}}{\#\ \mathrm{Electrons}} = \frac{\mathrm{sum}\left(\frac{EL_{\mathrm{raw}}(\lambda) - background(\lambda)}{t_{\mathrm{int}} \cdot \eta_{\mathrm{collect}} \cdot \eta_{\mathrm{transfer}}(\lambda)}\right)}{I/e}, \tag{S1}$$

where the $EL_{\mathrm{raw}}(\lambda)$ refer to the spectra as measured and the $background(\lambda)$ was recorded in the same condition without bias. The summed wavelength range is $700\,\mathrm{nm} - 900\,\mathrm{nm}$. t_{int} is the integration time of the spectra, η_{collect} is the collection efficiency of the oil objective, and $\eta_{\mathrm{transfer}}(\lambda)$ is the wavelength-dependent system transfer function of the detection path, from the entry of the oil-immersion objective to the collected data. I is the measured electrical current and e is the elementary charge.

B. Correction for the detection path

Due to losses introduced by lenses and the conversion efficiency of the spectrometer, the system transfer function of the detection path $\eta_{\mathrm{transfer}}(\lambda)$ has to be considered to determine the quantum efficiency of the device. To characterize the system transfer function of the detection path, a Halogen calibration lamp (Ocean Optics, HL-2000-CAL) with a known spectrum was used. Based on this, one could obtain a normalized transfer function. A reflection measurement with a 532 nm cw laser, focused on a gold surface was performed to calibrate the power losses throughout the beam path of the setup. This allowed us to get the absolute amplitude for the system transfer function to correct the light spectra and intensity.

However, except for entering the detection path at the glass side, the light emitted by the LED can also radiate in the opposite direction to the air, or propagate inside the h-BN slab until reaching the edges. Here we define the collection efficiency η_{collect} as the fraction we can detect with our oil objective directly from the device region. We exclude the light propagating along the h-BN that is out-scattered at the edges because it can be blocked spatially. A collection efficiency around 16.5 % (at ∼760 nm) was determined in a finite-difference time-domain (FDTD) simulation using a commercial software package (Lumerical Inc.). Here we note that this collection efficiency can vary a lot depending on the thickness of the encapsulating h-BN flakes. Two thinner encapsulating h-BN flakes (e.g. on the order of ∼50 nm) may lead to higher collection efficiency since the propagating slab mode in h-BN can hardly be supported.

C. Results

To calculate the external quantum efficiency of the LED, the photon number emitted by the device can be obtained by correcting the recorded spectra from the device region based on the analysis above. For a bias of $V_{\mathrm{b}} = 1.7\,\mathrm{V}$, the efficiency reaches ∼4 %.

IV. PHOTOLUMINESCENCE MEASUREMENTS

Figure S1a shows a representative PL (orange) spectrum from the device overlap when pumped with a laser (cw, 532 nm) at a photon energy higher than the optical bandgap. As expected, the PL emission is dominated by the neutral excitons of the WSe_2. The Fourier space image of the PL at the overlap region is shown in Fig. S1b which further indicates an emission that can be mainly attributed to in-plane oriented dipoles, as previously reported for excitons in monolayer TMDs [4]. When comparing the EL spectrum, shown in Fig. S1a (red), and the Fourier Space image (shown in the main text Fig. 5b) with the PL signals, the similarities confirm the identical origin of both emissions. The slight red-shift of the EL compared to PL has already been observed for devices with the same architecture and might be due to an increased trion contribution for the electrically driven emission [5, 6].

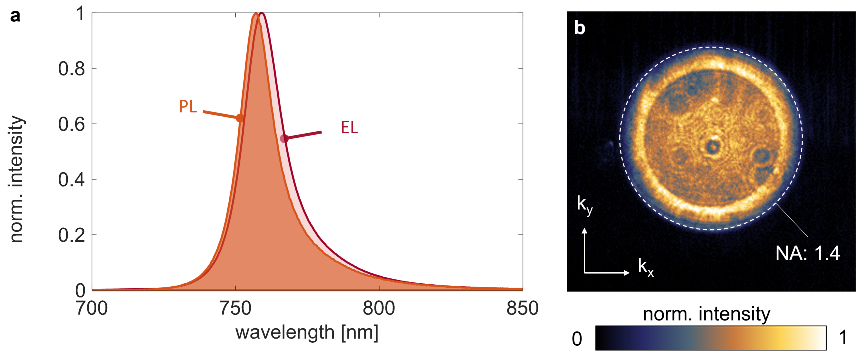

FIG. S1. (a) A normalized PL spectrum (orange) of the WSe_2 layer after stacking (from the overlap region), plotted together with the normalized EL spectrum (red) for an applied bias of $V_b = 1.7\,V$ after making edge contacts. (b) The Fourier Space image of PL.

V. GRAPHENE EDGE CONTACTS ON THICK STACKS

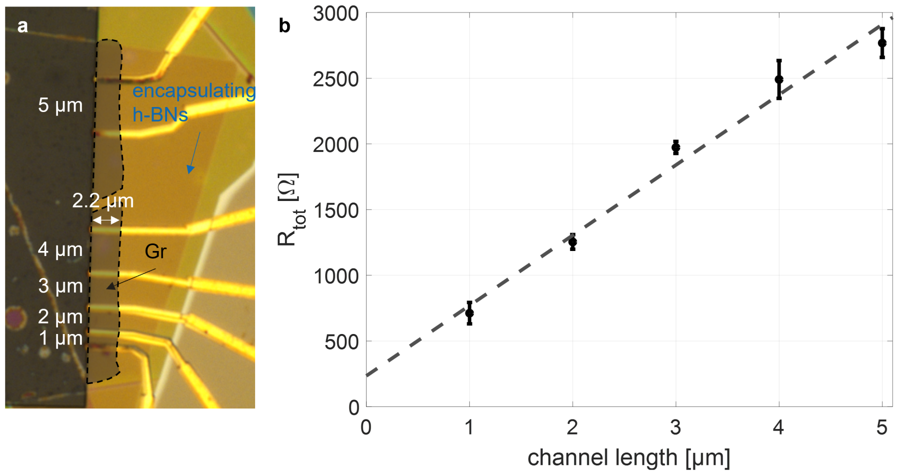

FIG. S2. (a) Optical microscope image of the reference stack for TLM measurements. The Gr sheet is indicated by the gray area. The channel lengths vary from 1 to 5 μm. The channel width is defined on one side by the edge of the Gr flake and on the other side by etching of the stack. (b) The total resistance of each channel, plotted together with a linear fit.

The contacting method of Gr edge contacts is a well-established approach for encapsulated structures [1, 3, 7]. However, since in our devices the encapsulating h-BNs are much thicker, in the following we characterize the edge contacts to Gr when integrated inside thick stacks. For this reason, we discuss transfer length method (TLM)

measurements on an h-BN – Gr – h-BN reference stack. The total stack thickness is ∼245 nm, whereby the Gr flake is integrated close to the vertical center. For the TLM characterization, Gr channels with lengths varying from 1 to 5 µm with a channel width of 2.2 µm (±0.2 µm) were defined (see Fig. S2a).

The total resistance for every channel can be described with

$$R_{\text{tot}} = 2R_{\text{met}} + 2R_{\text{C}} + R_{\text{sheet}} \frac{l_{\text{channel}}}{W}. \tag{S2}$$

R_{met} is the resistance of the metal trace, R_{C} is the contact resistance between Cr/Au and Gr, R_{sheet} is the sheet resistance of the Gr channel, l_{channel} and W are the channel length and width, respectively [7]. The total resistance of each channel is evaluated with I-V measurements and plotted in Fig. S2b. By a linear fit of the results (dashed line in Fig. S2b), the contact resistance can be determined, according to Eq. S2. The contact resistivity $\rho_{\text{C}} = R_{\text{C}} \cdot \text{W}$ can be extracted from the y-intercept. In our device the contact resistivity is 258 Ωµm ± 23 Ωµm, assuming a negligible metal resistance.

The assumption of a negligible resistance for the metal trace is correct for most devices [7]. In our devices, however, the resistance of the metal trace seems to have a non-negligible contribution to the total resistance. Reasons for this could be 1) the large step size from the remaining h-BN to the glass substrate and 2) the strong surface roughness of the glass after etching. From another reference device, with only a metal trace etched into h-BN, $2R_{\text{met}}$ is determined to be in the range of 50 – 100 Ω.

Overall, the TLM measurements help to estimate the contact resistance in our integrated LED structures. We conclude, that even when integrated inside thick h-BN flakes, the contact to the Gr electrodes is in a comparable order of magnitude as previously presented [1, 7].

VI. BAND ALIGNMENT OF THE TUNNELING JUNCTION

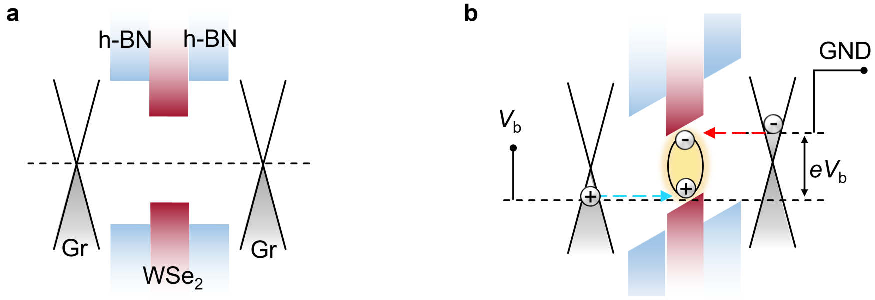

FIG. S3. (a) Band alignment of the tunneling junction without applying bias. (b) When the applied electrostatic potential eV_{b} is larger than the optical bandgap, carriers can tunnel into WSe_2 and form excitons [8].

VII. SPECTRAL CHARACTERISTICS OF THE WAVEGUIDE-COUPLED EMISSION

In the spectrum of the waveguide-coupled EL (see Fig. 5a in the main text), the main peak is positioned at ∼765 nm and a second smaller peak can be observed at ∼750 nm. Based on our data we cannot fully exclude the contribution of dark excitons to the waveguide-coupled emission. But as mentioned in the main text, the additional contribution of dark excitons is expected to be minor. Reasons for this are: 1) the oscillator strength is much weaker (∼1000 times) [9]. 2) The waveguide is designed such that only the fundamental transverse electric (TE) mode is supported. The dark exciton has an out-of-plane transition dipole [10, 11] and should therefore not couple efficiently to the TE mode of the waveguide.

Therefore, we now turn to the discussion of how the absorption of light by WSe_2 can affect the spectrum. To visualize this effect, we normalize the spectrum at the end of the waveguide to the spectrum at the LED region. $EL_{\text{WG}}(\lambda)/EL_{\text{LED}}(\lambda)$ is plotted in Fig. S4a for a voltage of 1.7 V (solid black line). Within the given wavelength range a distinct dip can be observed at around 755 nm, several nm below the free-space EL emission peak, plotted in red (in Fig. S4a). Such an abrupt dip has not been observed for simulations of a bare h-BN waveguide structure. This

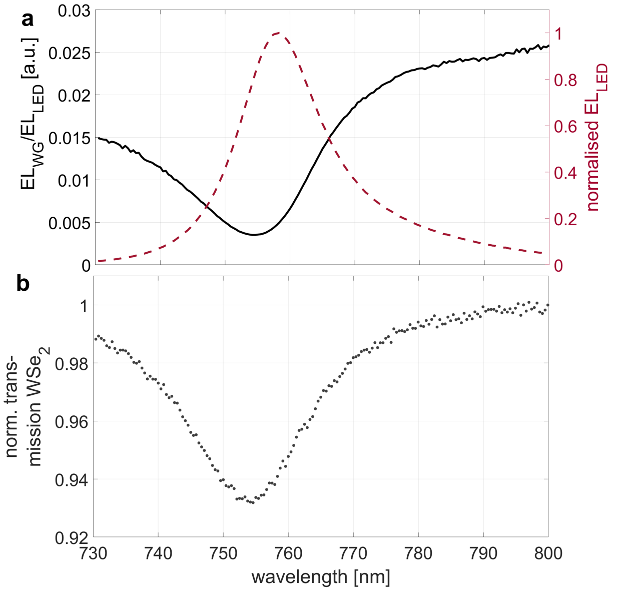

FIG. S4. (a) Waveguide-coupled EL normalized to the free-space EL (black solid line), visualizing the changes between the two spectra. For comparison, the free-space EL is plotted in red (dashed line). (b) Transmission spectrum of a WSe_2 monolayer encapsulated between two thin h-BN flakes. The sample was illuminated with a broadband source from one side and the transmitted light was collected from the other side using a second objective. The detected light was normalized to a reference spectrum obtained on the h-BN flakes.

is why we suggest that the spectral behavior can mainly be attributed to the optical properties of the incorporated monolayer.

For comparison, we visualize the absorption characteristics of a monolayer WSe_2 by plotting a free-space transmission spectrum in Fig. S4b. The monolayer (encapsulated by two thin h-BN flakes) was illuminated with a broadband source from one side and the transmitted light was detected from the other side with a second objective. Clearly, the spectral behavior of the integrated LED, plotted in Fig. S4a coincides with the free-space measured absorption characteristics of the monolayer (Fig. S4b). In earlier studies, the absorption has already been determined to be at wavelengths slightly shorter than the exciton PL emission [12]. Small deviations between the measurements in Figs. S4a and b can be explained by differences among the two monolayers (for transmission measurement: CVD grown flake). Note that the effect of absorption is especially pronounced in the integrated LED structure because the interaction length of the light with the part of the monolayer that is sticking out (see Fig. 4a in the main text) is in the µm range and not limited by the thickness of the flake. Such absorption effects have also been observed for confined whispering gallery modes [13] and surface plasmon polaritons [10].

VIII. DETAILS ON THE COUPLING EFFICIENCY

A. Experiments

The total coupling efficiency was evaluated from spectral integration (between $700-900$ nm) of the spatially resolved measurements, shown in Fig. 4c. The photon number detected at the waveguide end is divided by the photon number at the LED region. For a bias of 1.7 V, the uncorrected total coupling efficiency is 1.25 %. The emission properties from the LED region and from the waveguide end are different. Therefore, the collection efficiencies from both regions differ and corrections need to be taken into account.

The free-space EL from the LED, shown for the experiments in Fig. 5b and for simulations in Fig. S5a, is dominated by in-plane emitters oriented in the xy-plane. Because these emitters are integrated inside thick h-BN slabs, the majority of emission is, however, guided in the in-plane direction. Simulations for a single dipole, placed inside h-BN layers (identical top and bottom thickness as in the here presented device), show that for $\lambda = 760$ nm only $\sim$16.5 % are emitted into the glass substrate and collected by our oil immersion objective with an NA of 1.4 (same correction as discussed in section III).

The waveguide-coupled EL, on the other hand, is an in-plane guided mode, out-coupled through scattering at the

waveguide facet (cf. experimental measurement in Fig. 5c and simulations in Fig. S5b). From integration over the simulated intensity, within the collection cone of our objective, a collection efficiency of $\sim$70 % is determined. With the correction factors, from the simulations, the corrected total coupling efficiency is determined to be 0.3 % ($V_b = 1.7$ V). Note that when considering the wavelength-dependent coupling efficiency instead of the total coupling efficiency, in the spectral range of $730 - 800$ nm, it varies between 0.1 to 0.8 %. These wavelength-dependent variations and in general also other losses might be attributed to the wavelength-dependent absorption of the nearby monolayer WSe_2 and radiation losses due to the sidewall roughness.

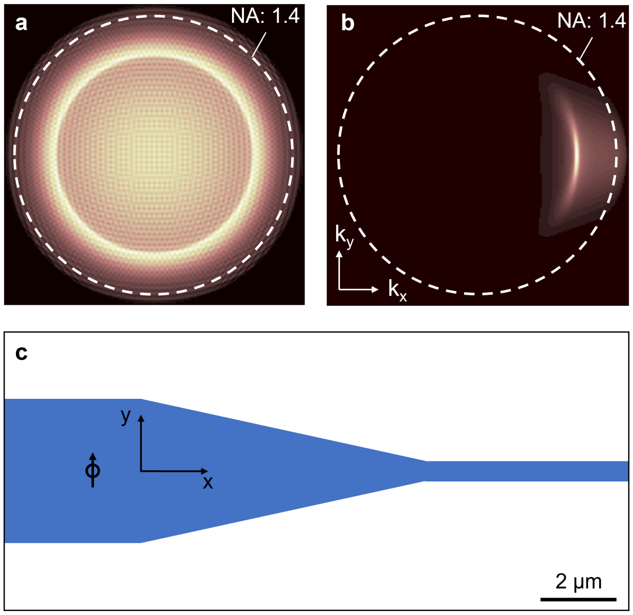

FIG. S5. (a) Simulated angular distribution (on the glass side) of in-plane dipoles (averaged over in-plane orientations) inside an h-BN slab. (b) Simulated angular distribution (on the glass side) of the fundamental TE mode (760 nm) at the end facet of the h-BN waveguide. (c) Sketch to illustrate the LED, tapering and waveguide as defined in the simulations.

B. Simulations

For the simulated coupling efficiency, a single dipole source was positioned inside an h-BN structure, as illustrated in Fig. S5c. The LED region, the tapering, and the waveguide were implemented according to the device geometry. The sidewall angle of 55° was determined from SEM images on a reference device and the refractive index of h-BN was taken for the given wavelength range from [14]. The waveguide is designed to only support fundamental TE modes (around the exciton wavelength of the WSe_2 monolayer). The determined coupling efficiency is therefore representing the ratio of the dipole emission that is coupled into the fundamental TE mode.

To furthermore understand the coupling efficiency from different regions throughout the LED region and for different dipole orientations within the xy-plane, the dipole position and the dipole angle (only for in-plane orientation) were swept independently. When sweeping the dipole in x-direction, only a small decrease is observed for an increased distance to the beginning of the tapering. However, when sweeping the position of the dipole in y-direction, the coupling efficiency decreases from 4.7 % to 0.3 % (x-position and angle of the dipole were still kept as shown in Fig. S5c). Similarly, for a dipole with an orientation along the x-axis the coupling efficiency decreases nearly to 0 % (x- and y-position of the dipole as shown in Fig. S5c). For the determined average (i.e. 1.2 %), the mean values of the individual sweeps were considered. It is therefore only a rough estimate. The coupling efficiency of the presented structure could be improved, by optimizing the lateral design of the LED region and the tapering.

Furthermore, numerical simulations for the integrated dipole emitter (at the same relative position as for the experimentally demonstrated LED) show a coupling efficiency $\sim$4 times higher compared to placing the emitter on top of the h-BN. This is in agreement with recent work on monolithically integrated h-BN emitters [15] and verifies the improved coupling efficiency when emitters are integrated inside the confinement layer at high field intensities. When considering z-dependent changes of the Purcell factor, the best coupling efficiency does not coincide perfectly

with the mode profile [16]. Adjustments in z-directions by around ∼10 nm might improve the coupling efficiency, however, only to a minor extent.

---

[1] Wang, L.; Meric, I.; Huang, P. Y.; Gao, Q.; Gao, Y.; Tran, H.; Taniguchi, T.; Watanabe, K.; Campos, L. M.; Muller, D. A.; Guo, J.; Kim, P.; Hone, J.; Shepard, K. L.; Dean, C. R. One-Dimensional Electrical Contact to a Two-Dimensional Material. *Science* **2013**, *342*, 614–617.

[2] Zomer, P. J.; Guimarães, M. H.; Brant, J. C.; Tombros, N.; Van Wees, B. J. Fast pick up technique for high quality heterostructures of bilayer graphene and hexagonal boron nitride. *Applied Physics Letters* **2014**, *105*, 013101.

[3] Overweg, H.; Eggimann, H.; Chen, X.; Slizovskiy, S.; Eich, M.; Pisoni, R.; Lee, Y.; Rickhaus, P.; Watanabe, K.; Taniguchi, T.; Fal'Ko, V.; Ihn, T.; Ensslin, K. Electrostatically Induced Quantum Point Contacts in Bilayer Graphene. *Nano Letters* **2018**, *18*, 553–559.

[4] Schuller, J. A.; Karaveli, S.; Schiros, T.; He, K.; Yang, S.; Kymissis, I.; Shan, J.; Zia, R. Orientation of luminescent excitons in layered nanomaterials. *Nature Nanotechnology* **2013**, *8*, 271–276.

[5] Withers, F.; Del Pozo-Zamudio, O.; Mishchenko, A.; Rooney, A. P.; Gholinia, A.; Watanabe, K.; Taniguchi, T.; Haigh, S. J.; Geim, A. K.; Tartakovskii, A. I.; Novoselov, K. S. Light-emitting diodes by band-structure engineering in van der Waals heterostructures. *Nature Materials* **2015**, *14*, 301–306.

[6] Withers, F. et al. WSe_2 Light-Emitting Tunneling Transistors with Enhanced Brightness at Room Temperature. *Nano Letters* **2015**, *15*, 8223–8228.

[7] Giubileo, F.; Di Bartolomeo, A. The role of contact resistance in graphene field-effect devices. *Progress in Surface Science* **2017**, *92*, 143–175.

[8] Binder, J.; Withers, F.; Molas, M. R.; Faugeras, C.; Nogajewski, K.; Watanabe, K.; Taniguchi, T.; Kozikov, A.; Geim, A. K.; Novoselov, K. S.; Potemski, M. Sub-bandgap Voltage Electroluminescence and Magneto-oscillations in a WSe_2 Light-Emitting van der Waals Heterostructure. *Nano Letters* **2017**, *17*, 1425–1430.

[9] Echeverry, J. P.; Urbaszek, B.; Amand, T.; Marie, X.; Gerber, I. C. Splitting between bright and dark excitons in transition metal dichalcogenide monolayers. *Phys. Rev. B* **2016**, *93*, 121107.

[10] Zhou, Y.; Scuri, G.; Wild, D. S.; High, A. A.; Dibos, A.; Jauregui, L. A.; Shu, C.; De Greve, K.; Pistunova, K.; Joe, A. Y.; Taniguchi, T.; Watanabe, K.; Kim, P.; Lukin, M. D.; Park, H. Probing dark excitons in atomically thin semiconductors via near-field coupling to surface plasmon polaritons. *Nature Nanotechnology* **2017**, *12*, 856–860.

[11] Zhang, X. X.; Cao, T.; Lu, Z.; Lin, Y. C.; Zhang, F.; Wang, Y.; Li, Z.; Hone, J. C.; Robinson, J. A.; Smirnov, D.; Louie, S. G.; Heinz, T. F. Magnetic brightening and control of dark excitons in monolayer WSe_2. *Nature Nanotechnology* **2017**, *12*, 883–888.

[12] Kozawa, D.; Kumar, R.; Carvalho, A.; Kumar Amara, K.; Zhao, W.; Wang, S.; Toh, M.; Ribeiro, R. M.; Castro Neto, A. H.; Matsuda, K.; Eda, G. Photocarrier relaxation pathway in two-dimensional semiconducting transition metal dichalcogenides. *Nature Communications* **2014**, *5*, 4543.

[13] Khelifa, R.; Back, P.; Flöry, N.; Nashashibi, S.; Malchow, K.; Taniguchi, T.; Watanabe, K.; Jain, A.; Novotny, L. Coupling Interlayer Excitons to Whispering Gallery Modes in van der Waals Heterostructures. *Nano Letters* **2020**, *20*, 6155–6161.

[14] Rah, Y.; Jin, Y.; Kim, S.; Yu, K. Optical analysis of the refractive index and birefringence of hexagonal boron nitride from the visible to near-infrared. *Opt. Lett.* **2019**, *44*, 3797–3800.

[15] Li, C.; Fröch, J. E.; Nonahal, M.; Tran, T. N.; Toth, M.; Kim, S.; Aharonovich, I. Integration of hBN Quantum Emitters in Monolithically Fabricated Waveguides. *ACS Photonics* **2021**, *8*, 2966–2972.

[16] Hoehne, T.; Schnauber, P.; Rodt, S.; Reitzenstein, S.; Burger, S. Numerical Investigation of Light Emission from Quantum Dots Embedded into On-Chip, Low-Index-Contrast Optical Waveguides. *Physica Status Solidi B* **2019**, *256*, 1800437.
